# Commentary: Healing the healer and changing the conditions

**DOI:** 10.1177/17449871261452645

**Published:** 2026-06-07

**Authors:** Pernilla Garmy

**Affiliations:** Professor, Kristianstad University, Sweden

**Commentary to:** Heal the healer: empowering healthcare providers to enact systems-level wellness changes. A mixed methods survey study (Poppert Cordts, 2025).

Accepting sleepless nights, a racing heart in the morning and hurried meals as part of everyday life. Skipping basic needs, even something as simple as going to the bathroom during a long shift.

This is not unusual. It is something I hear about again and again, whether in clinical settings, in academia or in conversations with colleagues. What should raise concern has simply become normal. At times it is even framed as a sign of commitment.

The study by Poppert Cordts et al. (2025) offers a timely contribution. The authors present a webinar-based intervention aimed at healthcare professionals, designed not only to strengthen individual coping but also to equip participants with the knowledge and skills needed to influence the systems that contribute to burnout. Their findings show increased knowledge, enhanced subjective competence and a clear intention among participants to apply what they have learned in practice. At the same time, a familiar pattern emerges. The barriers identified by participants are largely structural, including organisational hierarchies, financial constraints, staffing shortages and resistance to change.

This highlights a central tension in current approaches to workforce well-being. Even when burnout is recognised as a systems-level problem, the proposed solutions often remain focused on the individual.

In a recently published systematic review and meta-analysis, Jiménez-García and Flor-Martínez (2026) demonstrated that burnout among nurses can be reduced through targeted interventions. They found the strongest and most consistent evidence for mindfulness-based approaches and brief positive psychology interventions, particularly in reducing emotional exhaustion.

From my perspective as a nurse, academic and educator, workplace well-being is a critical issue. Universities and higher education institutions educate the future nursing workforce, generate knowledge about health and well-being and are themselves workplaces shaped by competing demands. High workloads, fragmented responsibilities and expectations of constant availability are often taken for granted. Here too, there is a risk that unhealthy working conditions become normalised, not through acute clinical pressure, but through a continuous accumulation of tasks related to teaching, research, supervision and administration.

In this context, I have personally sought ways to manage the demands of academic work. Drawing on literature on time and productivity, including the work of David Larsson Heidenblad (2023), I have experimented with strategies to create structure, prioritise and set boundaries. Regularly reviewing all ongoing commitments can make visible how many parallel processes compete for attention. Setting aside time to reflect on what is truly important, rather than simply urgent, can help redirect effort towards more meaningful work. At times, it may even be necessary to cancel commitments, step back and apologise, not as a sign of failure, but as a way to prevent long-term exhaustion.

These kinds of strategies can be helpful. They may restore a sense of control and create space for reflection. However, they also carry a risk. When presented as solutions to burnout, they can inadvertently shift attention away from the structural conditions that produce the problem in the first place.

There is nothing wrong with learning how to plan, prioritise and set limits. The problem arises when such skills become the organisation’s primary response to problems that the organisation itself has created.

The findings of Poppert Cordts et al. (2025) make this particularly clear. Participants expressed a strong willingness to improve communication, challenge harmful behaviours and contribute to more supportive workplace cultures. At the same time, they consistently pointed to barriers such as lack of managerial support, limited resources and deeply embedded cultural norms. This underscores that individual agency is always situated. It is shaped, and often constrained, by organisational structures.

The implications are therefore significant. Firstly, healthcare organisations need to recognise staff well-being as a central issue for quality of care and patient safety, not as a secondary or individual concern. Secondly, there is a need for structures that allow staff to act on the knowledge and motivation that interventions like this seek to generate. This includes not only time, resources and authority but also leadership that actively invites and supports staff engagement in change processes. Thirdly, nursing education should integrate both individual skills and organisational understanding, so that future nurses are equipped not only to manage stress but also to analyse and influence the systems in which they work.

Finally, the study points to the importance of combining approaches. Initiatives that empower individuals from the ground up are valuable, but they must be supported by organisational commitment from the top down. Without such alignment, even well-designed interventions risk remaining at the level of intention.
